# Quantitative SARS-CoV-2 anti-spike responses to Pfizer–BioNTech and Oxford–AstraZeneca vaccines by previous infection status

**DOI:** 10.1016/j.cmi.2021.05.041

**Published:** 2021-10

**Authors:** David W. Eyre, Sheila F. Lumley, Jia Wei, Stuart Cox, Tim James, Anita Justice, Gerald Jesuthasan, Denise O'Donnell, Alison Howarth, Stephanie B. Hatch, Brian D. Marsden, E. Yvonne Jones, David I. Stuart, Daniel Ebner, Sarah Hoosdally, Derrick W. Crook, Tim E.A. Peto, Timothy M. Walker, Nicole E. Stoesser, Philippa C. Matthews, Koen B. Pouwels, A. Sarah Walker, Katie Jeffery

**Affiliations:** 1) Oxford University Hospitals NHS Foundation Trust, Oxford, UK; 2) Nuffield Department of Population Health, University of Oxford, Oxford, UK; 3) NIHR Oxford Biomedical Research Centre, University of Oxford, Oxford, UK; 4) NIHR Health Protection Research Unit in Healthcare Associated Infections and Antimicrobial Resistance at University of Oxford in Partnership with Public Health England, Oxford, UK; 5) Nuffield Department of Medicine, University of Oxford, Oxford, UK; 6) Kennedy Institute of Rheumatology Research, University of Oxford, UK; 7) Target Discovery Institute, University of Oxford, Oxford, UK; 8) Oxford University Clinical Research Unit, Ho Chi Minh City, Viet nam

**Keywords:** Antibody, Quantitative anti-spike antibody, SARS-CoV-2, Serology, Vaccine

## Abstract

**Objectives:**

We investigated determinants of severe acute respiratory syndrome coronavirus 2 (SARS-CoV-2) anti-spike IgG responses in healthcare workers (HCWs) following one or two doses of Pfizer–BioNTech or Oxford–AstraZeneca vaccines.

**Methods:**

HCWs participating in regular SARS-CoV-2 PCR and antibody testing were invited for serological testing prior to first and second vaccination, and 4 weeks post-vaccination if receiving a 12-week dosing interval. Quantitative post-vaccination anti-spike antibody responses were measured using the Abbott SARS-CoV-2 IgG II Quant assay (detection threshold: ≥50 AU/mL). We used multivariable logistic regression to identify predictors of seropositivity and generalized additive models to track antibody responses over time.

**Results:**

3570/3610 HCWs (98.9%) were seropositive >14 days post first vaccination and prior to second vaccination: 2706/2720 (99.5%) were seropositive after the Pfizer–BioNTech and 864/890 (97.1%) following the Oxford–AstraZeneca vaccines. Previously infected and younger HCWs were more likely to test seropositive post first vaccination, with no evidence of differences by sex or ethnicity. All 470 HCWs tested >14 days after the second vaccination were seropositive. Quantitative antibody responses were higher after previous infection: median (IQR) >21 days post first Pfizer–BioNTech 14 604 (7644–22 291) AU/mL versus 1028 (564–1985) AU/mL without prior infection (p < 0.001). Oxford–AstraZeneca vaccine recipients had lower readings post first dose than Pfizer–BioNTech recipients, with and without previous infection, 10 095 (5354–17 096) and 435 (203–962) AU/mL respectively (both p < 0.001 versus Pfizer–BioNTech). Antibody responses >21 days post second Pfizer vaccination in those not previously infected, 10 058 (6408–15 582) AU/mL, were similar to those after prior infection followed by one vaccine dose.

**Conclusions:**

SARS-CoV-2 vaccination leads to detectable anti-spike antibodies in nearly all adult HCWs. Whether differences in response impact vaccine efficacy needs further study.

## Introduction

As vaccines against severe acute respiratory syndrome coronavirus 2 (SARS-CoV-2) are rolled out globally, individuals and their clinicians may wish to seek reassurance that vaccination has been ‘effective’ and to understand how long protection is likely to last. Given that current vaccines generate an immune response to viral spike antigens, anti-spike antibody titres, associated with neutralizing activity [[1], [2], [3]], provide a potential surrogate marker of protection. Therefore, understanding the assay- and time-dependent dynamics of post-vaccination anti-spike antibody measurements, how they differ between individuals (e.g. by age, gender, ethnicity, and with comorbidities), and how these findings relate to protection are increasingly important.

Multiple vaccines have been developed globally. In the UK, three vaccines have been approved for use [4], with Pfizer–BioNTech BNT162b2 and Oxford–AstraZeneca ChAdOx1 nCoV-19 (AZD1222) the most widely used; to date many individuals have received only one dose following an extension of the dosing interval to 12 weeks to maximize initial population coverage. The dynamics and magnitude of the immune response seen in vaccine immunogenicity trials are assay-dependent, predominantly focus on individuals without previous SARS-CoV-2 infection, and use in-house assays developed early in the pandemic rather than the commercially available, validated assays now accessible to diagnostic laboratories [1,[5], [6], [7], [8]]. Emerging real-world data show that nearly all individuals vaccinated with the mRNA vaccines Pfizer–BioNTech BNT162b2 and Moderna mRNA-1273 seroconvert by 21 days post first vaccine dose, with more rapid seroconversion and higher antibody titres seen in individuals previously infected with SARS-CoV-2 (using in-house ELISAs, and two commercial platforms) [[9], [10], [11], [12]]. Fewer immunogenicity data or comparative data for the Oxford–AstraZeneca vaccine are available outside of clinical trials. No trials have published data on whether measured immune markers correspond to observed vaccine efficacy (i.e. protection from infection, hospitalization or death).

Here we compare anti-spike IgG responses, using a widely-available commercial assay, in healthcare workers (HCWs) following one or two vaccine doses with either the Pfizer–BioNTech or the Oxford–AstraZeneca vaccines. We also assess how responses vary between those with and those without previous evidence of infection.

## Methods

### Setting

Post-vaccination antibody responses were studied in HCWs from Oxford University Hospitals (OUH), four teaching hospitals in Oxfordshire, UK. Data on previous infections were available from symptomatic testing offered to HCWs with new persistent cough, fever ≥37.8°C or anosmia/ageusia from 27th March 2020 by OUH and from community-based PCR-positive test results shared with the hospital by public health agencies and HCWs. In addition, asymptomatic HCWs were offered voluntary nasal and oropharyngeal swab PCR testing every 2 weeks and serological testing every 2 months from 23rd April 2020 [[13], [14], [15]]. Staff were encouraged to attend for serological testing prior to first and second vaccinations, and additionally around 4 weeks post first vaccination where the second vaccine dose was due to be given after 12 weeks.

The staff vaccination programme began on 8th December 2020, starting with the Pfizer–BioNTech BNT162b2 vaccine, with Oxford–AstraZeneca ChAdOx1 nCoV-19 added from 4th January 2021 and predominately provided to staff at one acute hospital. Some HCWs received the Oxford–AstraZeneca vaccine in clinical trials beginning 23rd April 2020 and were included following unblinding if receiving active vaccine.

### Laboratory assays

PCR tests were performed by the OUH and community test centres using a range of assays (see Supplementary Material). Post-vaccination anti-spike IgG responses were assessed using the Abbott SARS-CoV-2 IgG II Quant antibody test targeting the spike receptor binding domain (RBD), with results available up to 11th March 2021. The assay cut-off is ≥ 50 AU/mL, with linear quantification of detected results from 50 to 40 000 AU/mL reported by the manufacturer (and confirmed by serial dilution of monoclonal antibodies: Supplementary Material Fig. S1).

Pre-vaccination antibody status was assessed using the Abbott anti-nucleocapsid IgG assay (defining readings of ≥1.4 as detected), the Abbott anti-spike IgG, and an anti-trimeric spike IgG ELISA [16] (detected: ≥8 million units).

### Statistical analysis

Staff were grouped into those with evidence of prior infection—i.e. any positive anti-spike or anti-nucleocapsid antibody test or positive PCR prior to first vaccination—and those without (including staff with no previous serology or PCR testing). Proportions that were anti-spike-positive were estimated by week post first vaccination, censoring follow-up at the second vaccination, and by week post second vaccination.

We used multivariable logistic regression to identify predictors of any positive anti-spike antibody result ≥15 days post first vaccination (but before a second vaccination), considering the vaccine given, previous infection status, age, sex, and ethnicity. We modelled quantitative antibody titres by day since first and second vaccination using generalized additive models, adjusting for age and fitting separate models by vaccine and prior infection status (details in the Supplementary Material).

### Ethics statement

Deidentified data were obtained from the Infections in Oxfordshire Research Database which has generic Research Ethics Committee, Health Research Authority and Confidentiality Advisory Group approvals (19/SC/0403, 19/CAG/0144).

## Results

Post-vaccination quantitative anti-spike results were available following either a first or a second dose or both for 4315 HCWs; 3377 antibody measurements were available (in 2863 HCWs) following a first dose of Pfizer–BioNTech and 1108 (992 HCWs) following a first dose of Oxford–AstraZeneca vaccine. The demographics and occupational roles of those receiving the Pfizer–BioNTech or Oxford–AstraZeneca were broadly similar; a slightly lower proportion of Pfizer–BioNTech recipients were female (81% versus 85%) and median ages were similar (41 versus 42 years) (Supplementary Material Table S1). Following a second dose of each vaccine, 560 (483 HCWs) and 25 (21 HCWs) anti-spike results respectively were available: median (IQR) dosing interval 24 (21–28) days.

### Antibody positivity post first and second vaccination

**Fig. 1:**
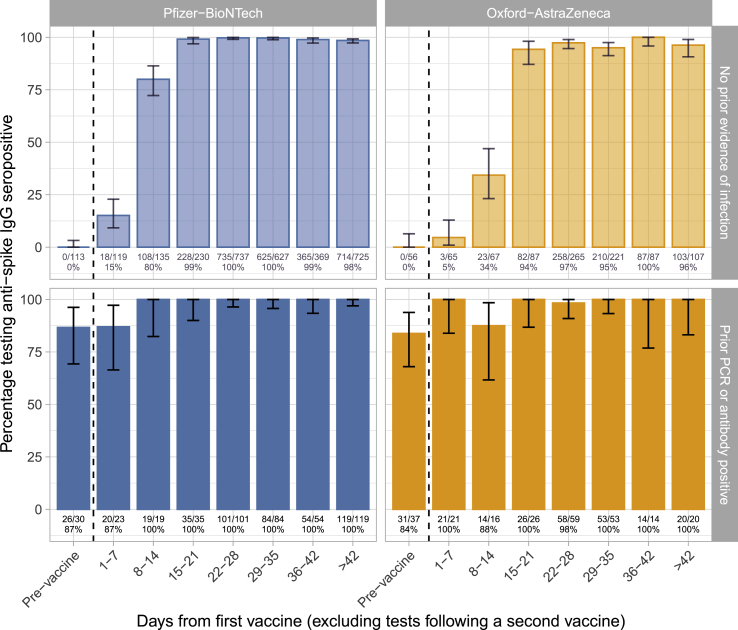
Anti-spike IgG-positive results by days since first vaccination, by prior infection status and vaccine received. Tests performed after a second dose of vaccine are not included. The number of tests performed and positive and the resulting percentage is shown under each bar.

Anti-spike antibody responses rose in the 14 days post first vaccination, such that from day 15 onward nearly 100% seroconversion was seen regardless of vaccine received or previous infection status (Fig. 1). Overall, 3570/3610 HCWs (98.9%) were seropositive when tested >14 days post first vaccination and prior to second vaccination, 2706/2720 (99.5%) of those receiving the Pfizer–BioNTech vaccine versus 864/890 (97.1%) receiving the Oxford–AstraZeneca vaccine.

**Fig. 2:**
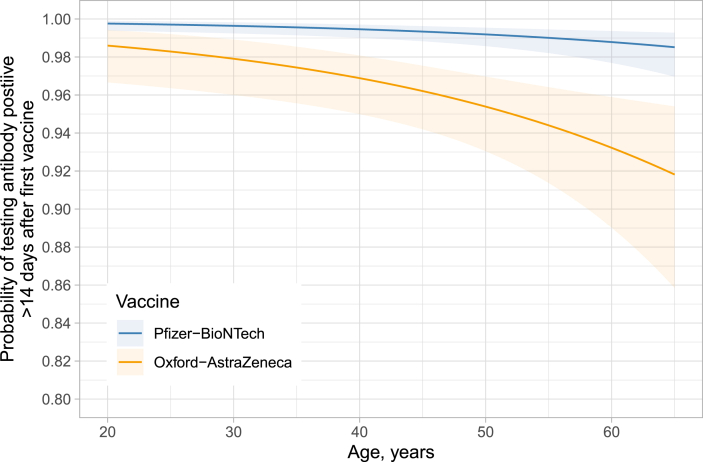
The relationship between vaccine, age and probability of testing anti-spike IgG seropositive >14 days post first vaccination. Model predictions are shown using reference categories for sex and ethnicity (white, female, respectively) and in those without prior evidence of infection.

In a regression model of responses >14 days post first vaccination and before second vaccination (Table 1), previously infected HCWs were independently more likely to test seropositive (in part reflecting that previously positive serology was one of the criteria for assessing previous infection: adjusted odds ratio (aOR) 6.99, 95%CI 0.95–51.3, p 0.06) and older HCWs were less likely (aOR per 10 years older 0.66, 95%CI 0.51–0.86, p 0.002). There was no evidence of an effect of sex or ethnicity. Oxford–AstraZeneca vaccine recipients were less likely than Pfizer–BioNTech recipients to seroconvert after their first vaccine dose (aOR 0.17, 95%CI 0.09–0.33, p < 0.001). However, the absolute probability of seroconversion remained near 100% across most groups, with the exception of older HCWs receiving the Oxford–AstraZeneca vaccine: e.g. a 60-year-old white female HCW had a 98.8% (95%CI 97.7–99.4%) chance of seroconversion with Pfizer–BioNTech and 93.2% (95%CI 89.0–95.9%) with Oxford–AstraZeneca post first dose (Fig. 2, Supplementary Material Fig. S2).

**Table 1:** Factors associated with seropositivity ≥15 days post first vaccination: univariable and multivariable logistic regression^a^

Variable	Summary		Univariable			Multivariable		
	Anti-spike IgG not detected, *n* = 40^b^	Anti-spike IgG detected, *n* = 3570^b^	Unadjusted odds ratio	95%CI	p value	Adjusted odds ratio	95%CI	p value
Previous infection								
No prior evidence of infection	39 (1.3%)	3070 (99%)	—	—		—	—	
Prior PCR or antibody positive	1 (0.2%)	500 (100%)	6.22	0.85, 45.4	0.071	6.99	0.95, 51.3	0.056
Vaccine								
Pfizer-BioNTech	14 (0.5%)	2706 (99%)	—	—		—	—	
Oxford-AstraZeneca	26 (2.9%)	864 (97%)	0.17	0.09, 0.33	<0.001	0.17	0.09, 0.33	<0.001
Sex								
Female	33 (1.1%)	2921 (99%)	—	—		—	—	
Male	7 (1.1%)	648 (99%)	1.01	0.44, 2.28	0.99	0.77	0.33, 1.79	0.55
Prefer not to say	0 (0%)	1 (100%)						
Ethnic group								
White	37 (1.3%)	2774 (99%)	—	—		—	—	
Asian	2 (0.4%)	480 (100%)	3.20	0.77, 13.3	0.11	2.50	0.59, 10.6	0.21
Black	1 (1.1%)	91 (99%)	1.21	0.16, 8.95	0.85	1.34	0.18, 10.0	0.78
Other	0 (0%)	225 (100%)						
Age	49 (39, 58)	41 (30, 51)						
Age, per 10 year older			0.64	0.49, 0.83	<0.001	0.66	0.51, 0.86	0.002

a Test results obtained after a second vaccination are not included. Those of ‘Other’ ethnicity or non-disclosed sex are omitted from the regression models as all tested seropositive. Of 501 healthcare workers (HCWs) with evidence of prior infection, 246 had a prior positive PCR result (median (IQR) 235 (46–263) days before first vaccination); of the remainder, 168 had detected anti-nucleocapsid IgG antibodies and 229 detected anti-spike IgG antibodies prior to vaccination (first detected: median (IQR) 231 (99–248) days prior to first vaccination).

b *n* (%); median (IQR).

All 448 HCWs with an antibody test >14 days after their second Pfizer–BioNTech vaccine were seropositive. Relatively few HCWs were vaccinated twice with the Oxford–AstraZeneca vaccine, but all 22 assayed >14 days post second dose were seropositive (Supplementary Material Fig. S3).

### Quantitative antibody readings before and after vaccination

Pre-vaccination quantitative antibody levels were available in 67 previously infected HCWs and 169 without evidence of prior infection; median (IQR) readings were 334 (103–1070) and 0.1 (0–1.4) AU/mL respectively. The median (IQR) time from first evidence of previous infection (first positive PCR or serological test) in those previously infected was 31 (0–246) days, with no evidence of association with antibody levels (Spearman's ρ = –0.09, p 0.45; Supplementary Material Fig. S4).

**Fig. 3:**
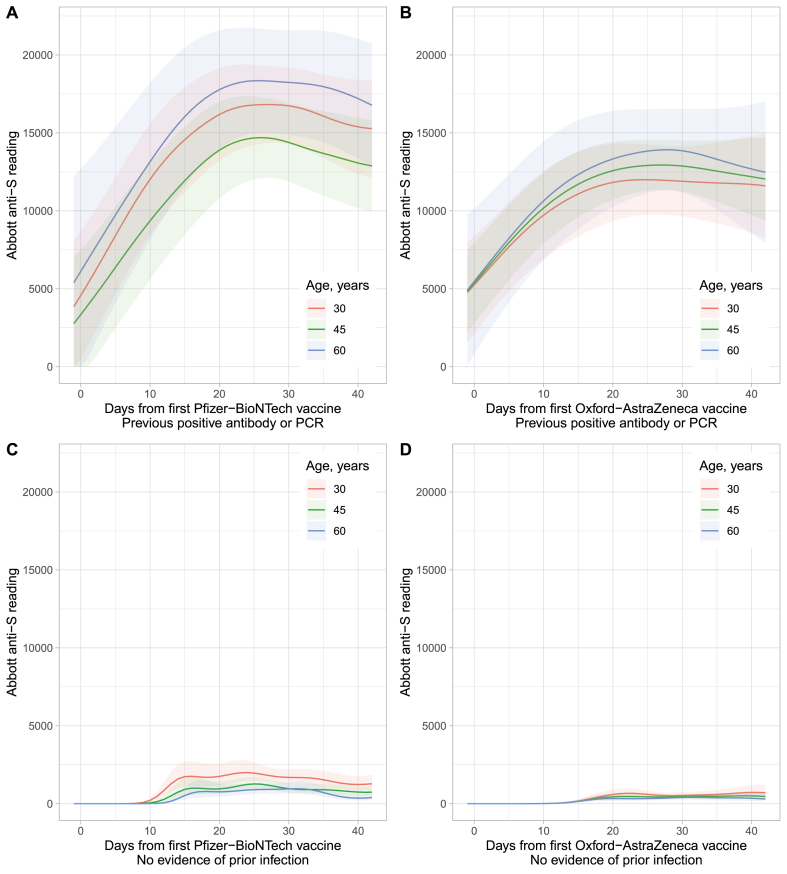
Modelled quantitative anti-spike IgG responses following first vaccination by vaccine and previous infection status. Panels A and B show responses in previously infected healthcare workers (HCWs) and panels C and D HCWs without evidence of previous infection. Panels A and C show data for those receiving Pfizer–BioNTech vaccine and panels B and D Oxford–AstraZeneca vaccine. Model predictions are shown at three example ages: 30, 45, and 60 years. The shaded ribbon shows the 95% confidence interval. Values are plotted from 7 days prior to vaccination to illustrate baseline values (models are fitted using data from 28 days prior to vaccination onwards).

Quantitative vaccine readings rose during the 3 weeks post first vaccination before plateauing (Fig. 3). Those with previous infection developed substantially higher titres. In those receiving the Pfizer–BioNTech vaccine, the median (IQR) anti-spike IgG reading >21 days post first vaccine dose was 1028 (564–1985) AU/mL without evidence of prior infection and 14 604 (7644–22 291) AU/mL with (Kruskal–Wallis p < 0.001). Those receiving the AstraZeneca vaccine had lower titres compared to the Pfizer–BioNTech, without and with previous infection 435 (203–962) AU/mL and 10 095 (5354–17 096) AU/mL respectively (p < 0.001 versus Pfizer–BioNTech and within AstraZeneca). In previously uninfected HCWs, after Pfizer–BioNTech vaccination higher titres were seen in younger age groups (Fig. 3C). Otherwise, there was no clear relationship between age and post-vaccination antibody readings.

**Fig. 4:**
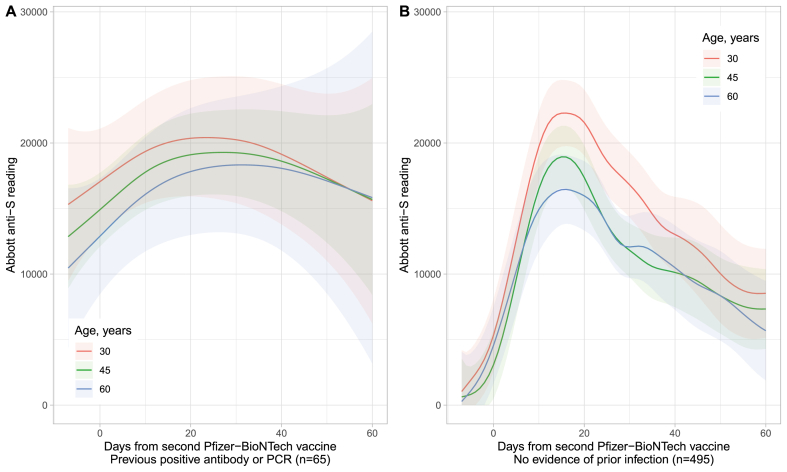
Modelled quantitative anti-spike IgG titres following second Pfizer–BioNTech vaccination by previous infection status. Panel A shows those who were previous infected (including those previously infected at baseline or testing PCR-positive between vaccines) and panel B those who had no evidence of previous infection. Model predictions are shown at three example ages: 30, 45, and 60 years. The shaded ribbon shows the 95% confidence interval. Data were included in each model from 7 days before the second vaccination to allow pre-vaccination levels to be fitted correctly.

In HCWs receiving a second Pfizer–BioNTech vaccine dose, antibodies were boosted in previously uninfected individuals, with the highest levels in younger HCWs, but with some waning of responses from day 20 to 60 post vaccination (Fig. 4). Median (IQR) anti-spike IgG readings >21 days post second vaccine dose were 10 058 (6408–15 582) AU/mL without evidence of previous infection and 18 047 (10 884–22 413) AU/mL with such evidence. Hence, anti-spike readings post second vaccination in those without evidence of previous infection (Fig. 4B) were similar to those seen after one vaccination in previously infected HCWs (Figs. 3A,B).

### Infections post vaccination

Of 4069 HCWs who had an anti-spike IgG measurement >14 days after first vaccination, including those assessed after a second vaccination, only eight HCWs had a subsequent positive PCR test after their antibody test (median (range) 47 (37–70) days post first vaccination). Therefore, there was very limited power to detect any relationship between protection and quantitative antibody readings. The median (IQR) (range) maximum post vaccination, pre-infection antibody measurement in those infected was 1004 (733–1958) (644–8151) AU/mL and 1361 (580–4612) (0–40 000) AU/mL in those not (Kruskal–Wallis p 0.78).

## Discussion

In this cohort of >4000 HCWs, predominantly healthy adults of working age, 98.9% developed a positive anti-spike IgG antibody test by > 14 days post-first vaccination. HCWs in their 50s and 60s were less likely to seroconvert than younger HCWs, but absolute rates of seroconversion remained high in all groups. Fewer HCWs were seropositive after a first dose of Oxford–AstraZeneca compared to Pfizer–BioNTech vaccine, with the difference more marked as age increased. Following a first dose, Pfizer–BioNTech vaccine resulted in higher antibody readings than the Oxford–AstraZeneca vaccine in both those with and those without previous infection, over two-fold higher if not previously infected. All HCWs assessed >14 days post second vaccination tested seropositive, although this included only 22 Oxford–AstraZeneca recipients.

The biggest determinant of the magnitude of quantitative antibody responses post first vaccine dose was previous infection, with median readings more than ten-fold higher with previous infection for both vaccines compared to without previous infection. Antibody levels after prior infection and a single vaccine dose were similar to those achieved after two vaccine doses. Taken together with evidence that natural infection without vaccination offers similar protection from infection to two doses of vaccine [17], these data support prioritizing uninfected individuals where vaccine-sparing strategies are required. It may be possible to delay the first vaccination in previously infected healthy individuals, and studies to assess whether one dose of vaccine may be sufficient where there is robust serological evidence of previous infection could be considered.

We found lower rates of seroconversion in older HCWs (~60 years). The Pfizer–BioNTech trial reported that neutralizing titres and S1-binding antibody concentrations were higher in younger (18–55 years) versus older (56–85 years) participants [6]; however, high seroconversion rates by S-binding antibodies were observed in Oxford–AstraZeneca trials involving older adults (≥65 years) after their first (97.3%, *n* = 149, 95%CI 93.3–99.3) and second doses (100.0%, *n* = 156, 95%CI 97.7–100.0) [8].

Although we observed differences in the proportion seroconverting and the magnitude of the response following Pfizer–BioNTech and Oxford–AstraZeneca vaccines, this should not be taken alone as evidence that one vaccine is likely to be more efficacious than the other. Data from the UK's national COVID Infection Survey demonstrate similar vaccine efficacy following a first dose of both Pfizer–BioNTech and Oxford–AstraZeneca vaccines [17].

Anti-spike antibody titres are associated with neutralizing activity [[1], [2], [3]], but the degree to which binary or qualitative anti-spike results are a surrogate for protection against infection, or other endpoints of interest such as hospitalization, death, or onward transmission, remains unclear. Our study was insufficiently powered to determine the relationship between antibody titres and protection, with only eight HCWs infected after a post-vaccine antibody test. However, all eight had positive antibody results with readings ranging from 644 to 8151 AU/mL, such that much of the range of positive anti-spike readings recorded in the study is not associated with total protection from infection. Although nearly all staff seroconverted post first dose, we have previously shown single-dose vaccine effectiveness against any positive PCR test to be 64% in this cohort of HCWs, and 90% following two doses [18]. This discrepancy between seroconversion and protection against reinfection, particularly post first dose, is similar to that reported in Oxford–AstraZeneca trial participants; 98.5% seroconverted 28 days post first dose, but pooled vaccine efficacy post first dose was 76.0% (95%CI 59.3–86.9%) [19]. Seroconversion rates are not reported in the Pfizer–BioNTech trial. This discrepancy may in part be because the protective anti-spike threshold may differ from the assay positive/negative threshold, and also due to variation in protection mediated by other mechanisms. Further large-scale studies will be required to determine whether quantitative antibody levels can act as a surrogate marker for protection. Antibody testing post vaccination may also play other roles. It may be useful to identify individuals in high-risk groups who do not seroconvert [20], who may remain at higher risk of infection, and may benefit from tailored advice around social contact, and, depending on underlying comorbidity, from further vaccination doses e.g. if immune reconstitution is expected.

Limitations of this study include the use of a single assay to quantify post-vaccination anti-spike antibody levels; however, as it is commercially-available and well calibrated (Supplementary Material Fig. S1), results should be generalizable. Our focus on a defined group (HCWs) is both a strength and a weakness, and we are not able to assess variations in vaccine response in children or those >65 years. Additional data on post-vaccination antibody responses in older individuals, at highest risk of adverse outcomes from SARS-CoV-2 infection, is particularly important. Our cohort was also 76% female and predominantly of white ethnicity. We did not assess neutralizing antibodies or T-cell responses, both of which reflect vaccine response and may vary by vaccine. However, as the anti-spike assay used targets the RBD of SARS-CoV-2, quantitative antibody readings are likely to be indicative of neutralizing activity [[1], [2], [3]]. Studying T-cell responses at scale is more logistically challenging but is the subject of several ongoing studies, such as the PITCH study in UK healthcare workers. Further work will be required to determine the duration of antibody responses and to compare antibody responses following two doses of Oxford–AstraZeneca (underrepresented in this current study) with two doses of Pfizer–BioNTech vaccine.

In summary, vaccination leads to detectable anti-spike antibodies in nearly all healthy adult HCWs. Markedly higher responses to vaccine are seen after previous infection; single dose or delayed vaccination could be considered where vaccine sparing is needed in healthy individuals with robust evidence of previous infection. Some caution is required with interpretation of antibody results and any subsequent behaviour change, as despite good protection from vaccination, seroconversion with high antibody levels still does not afford absolute protection from infection. Large-scale studies will be required to assess how protection from infection varies by antibody titre.

## Author contributions

Conceptualization: DWE, SFL, DWC, TEAP, TMW, NEW, PCM, KBP, ASW and KJ. Methodology: DWE, JW, KBP and ASW. Formal analysis: DWE. Investigation: SC, TJ, AJ, GJ, DOD, AH, SBH, BDM and DE. Writing – original draft: DWE and SFL. Writing – review & editing: all authors. Visualization: DWE. Supervision: DWE, EYJ, DIS, DE, SH, DWC, TEAP, TMW, ASW and KJ. Project administration: SH.

## Transparency declaration

DWE declares lecture fees from 10.13039/100016016Gilead, outside the submitted work. No other author has a conflict of interest to declare. DWE is a Robertson Foundation Fellow and an NIHR Oxford BRC Senior Fellow. SFL is a Wellcome Trust Clinical Research Fellow. DIS is supported by the Medical Research Council (MR/N00065X/1). PCM holds a Wellcome Intermediate Fellowship (110110/Z/15/Z). BDM is supported by the Kennedy Trust for Rheumatology Research. TMW is a Wellcome Trust Clinical Career Development Fellow (214560/Z/18/Z). ASW is an NIHR Senior Investigator. This work was supported by the UK Government's Department of Health and Social Care. This work was also supported by the National Institute for Health Research Health Protection Research Unit (NIHR HPRU) in Healthcare Associated Infections and Antimicrobial Resistance at Oxford University in partnership with Public Health England (PHE) (NIHR200915), the NIHR Biomedical Research Centre, Oxford, and benefactions from the Huo Family Foundation and Andrew Spokes. The views expressed in this publication are those of the authors and not necessarily those of the NHS, the National Institute for Health Research, the Department of Health, or Public Health England. This study is affiliated with Public Health England's SARS-CoV-2 Immunity & Reinfection EvaluatioN (SIREN) study.

## Access to data

The datasets analysed during the current study are not publicly available as they contain personal data but are available from the Infections in Oxfordshire Research Database (https://oxfordbrc.nihr.ac.uk/research-themes-overview/antimicrobial-resistance-and-modernising-microbiology/infections-in-oxfordshire-research-database-iord/), subject to an application and research proposal meeting the ethical and governance requirements of the Database.
