# Supplementary material for: Quantitative SARS-CoV-2 anti-spike responses to Pfizer–BioNTech and Oxford–AstraZeneca vaccines by previous infection status
Source: Clin Microbiol Infect. 2021 Oct;27(10):1516.e7–1516.e14. doi: 10.1016/j.cmi.2021.05.041 (PMC8180449; doi:10.1016/j.cmi.2021.05.041)
Supplement: Multimedia component 1 [file mmc1.pdf]

Quantitative SARS-CoV-2 anti-spike responses to Pfizer-BioNTech and Oxford-AstraZeneca vaccines by previous infection status: Supplementary material

Supplementary methods

PCR assays

RT-PCR was performed using the Public Health England SARS-CoV-2 assay (targeting the RdRp gene), one of five commercial assays: Abbott RealTime (targeting RdRp and N genes; Abbott, Maidenhead, UK), Altona RealStar (targeting E and S genes; Altona Diagnostics, Liverpool, UK), Cepheid Xpert® Xpress SARS-CoV-2 (targeting N2 and E; Cepheid, California, USA), BioFire® Respiratory 2.1 (RP2.1) panel with SARS-CoV-2 (targeting ORF1ab and ORF8; Biofire diagnostics, Utah, USA), Thermo Fisher TaqPath assay (targeting S and N genes, and ORF1ab; Thermo Fisher, Abingdon, UK) or using the ABI 7500 platform (Thermo Fisher, Abingdon, UK) with the US Centers for Disease Control and Prevention Diagnostic Panel of two probes targeting the N gene.

PCR-positive results from community-based symptomatic testing of Oxford University Hospitals (OUH) healthcare workers (HCWs) forwarded by public health agencies were also included (Thermo Fisher TaqPath assay).

Serological assays

The manufacturer's reported sensitivity of the Abbott SARS-CoV-2 IgG II Quant antibody test is 157/158 (99.4%, 95%CI 96.5-99.9%) ≥15 days after symptom onset in individuals with PCR-confirmed SARS-CoV-2 infection. Reported specificity is 1999/2008 (99.6%, 95%CI 99.2-99.8%) (assay instructions for use).

Pre-vaccination antibody status was assessed using the Abbott anti-nucleocapsid IgG assay (defining readings of ≥1.4 as detected), the Abbott anti-spike IgG, and the Oxford anti-trimeric spike IgG ELISA (detected: ≥8 million units). The sensitivity and specificity of the Abbott anti-nucleocapsid assay has been previously evaluated as 92.7% (95% CI 90.2-94.8) and 99.9% (99.4-100%) and the sensitivity and specificity of the Oxford anti-spike assay as 99.1% (97.8-99.7) and 99.0% (98.1-99.5) respectively.[1]

Vaccinations

Staff vaccinations provided by the hospital were recorded on an electronic database linked to testing data; staff were also able to supply details of vaccinations from other providers when requesting a symptomatic or asymptomatic PCR test.

Statistical models

Analyses were undertaken using R (v.4.04) and the mgcv (v.1.8-34) and splines (v3.6.2) libraries. We used multivariable logistic regression to identify associations with a positive anti-spike antibody result at any time ≥15 days post-first vaccination (but before a second vaccination), considering the vaccine given, previous infection status, age, sex, and ethnicity. We allowed for non-linear effects of

age using natural cubic splines with up to 5 default spaced knots, choosing the best fitting model using Akaike information criterion. We checked for evidence of collinearity between model factors using the vif function from the car package (v3.0-10).

We modelled quantitative antibody titres by day since post-first vaccination using generalised additive models, adjusting for age. Separate models were fitted by previous infection status and for each of the two vaccines. To allow for estimation of baseline pre-vaccine antibody levels, antibody readings from up to 28 days prior to first vaccination were included. We allowed for non-linear main effects and interactions for time since vaccination and age using tensor product smoothers. We allowed for repeat measurements in the same individual by including a random effect smoother. Example code for running the analysis is provided below, where the basis dimensions k1 and k2 are chosen based on optimising model fit:

```
m = bam(abbott_spike_reading ~ te(days_from_vaccine1, age, bs="tp",
k=c(k1,k2))+ s(user_id, bs="re"), method = "REML", data=df)
```

Similar models were fitted following a second vaccine dose for those receiving the Pfizer-BioNTech vaccine. Insufficient data were available after a second Oxford-AstraZeneca vaccine to fit models. Results from up to 7 days before second vaccine were included to allow for estimation of levels at the time of second vaccination. Model diagnostics were checked using the gam.check function from the mgcv package. Models following first vaccination in those not previously infected were fitted using log transformed antibody readings as this resulted in more normally distributed model residuals.

References

1 National SARS-CoV-2 Serology Assay Evaluation Group. Performance characteristics of five immunoassays for SARS-CoV-2: a head-to-head benchmark comparison. *Lancet Infect. Dis.* (2020) doi:10.1016/S1473-3099(20)30634-4.

Supplementary figures

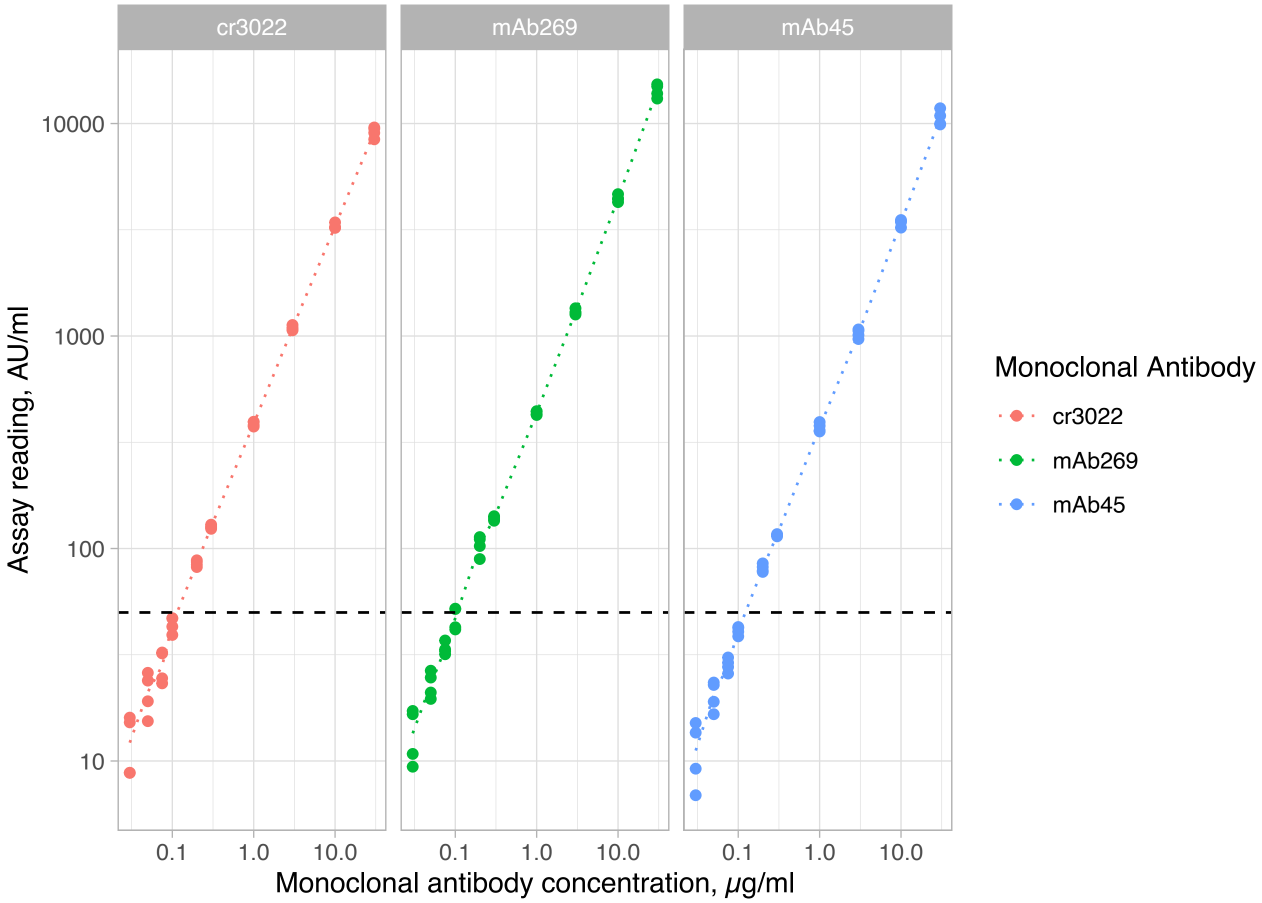

Figure S1. Abbott SARS-CoV-2 IgG II Quant antibody test calibration. A linear response is seen between the concentration of 3 SARS-CoV-2 anti-spike monoclonal antibodies (cr3022, mAb269, mAb45) and the assay reading. Both axes are shown on a log10 scale. The horizontal dashed line indicates the assay cut-off of ≥50 AU/ml. The dotted line for each assay joins the mean values at each dilution.

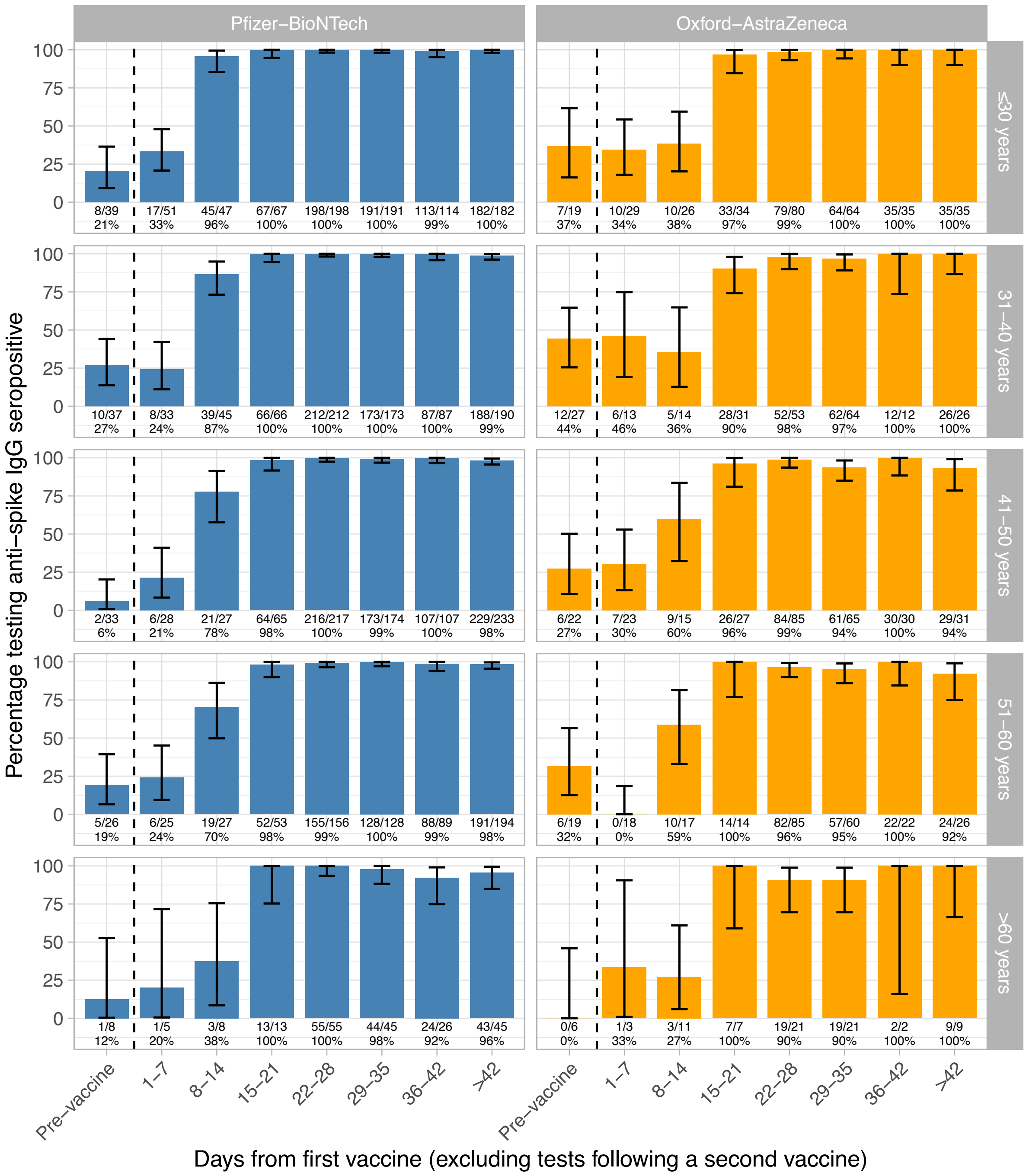

Figure S2. Anti-spike IgG positive results by days since first vaccination, by age group and vaccine received. The figure includes healthcare workers with and without evidence of prior infection. Tests performed after a second dose of vaccine are not included. The number of tests performed and positive and the resulting percentage is shown under each bar.

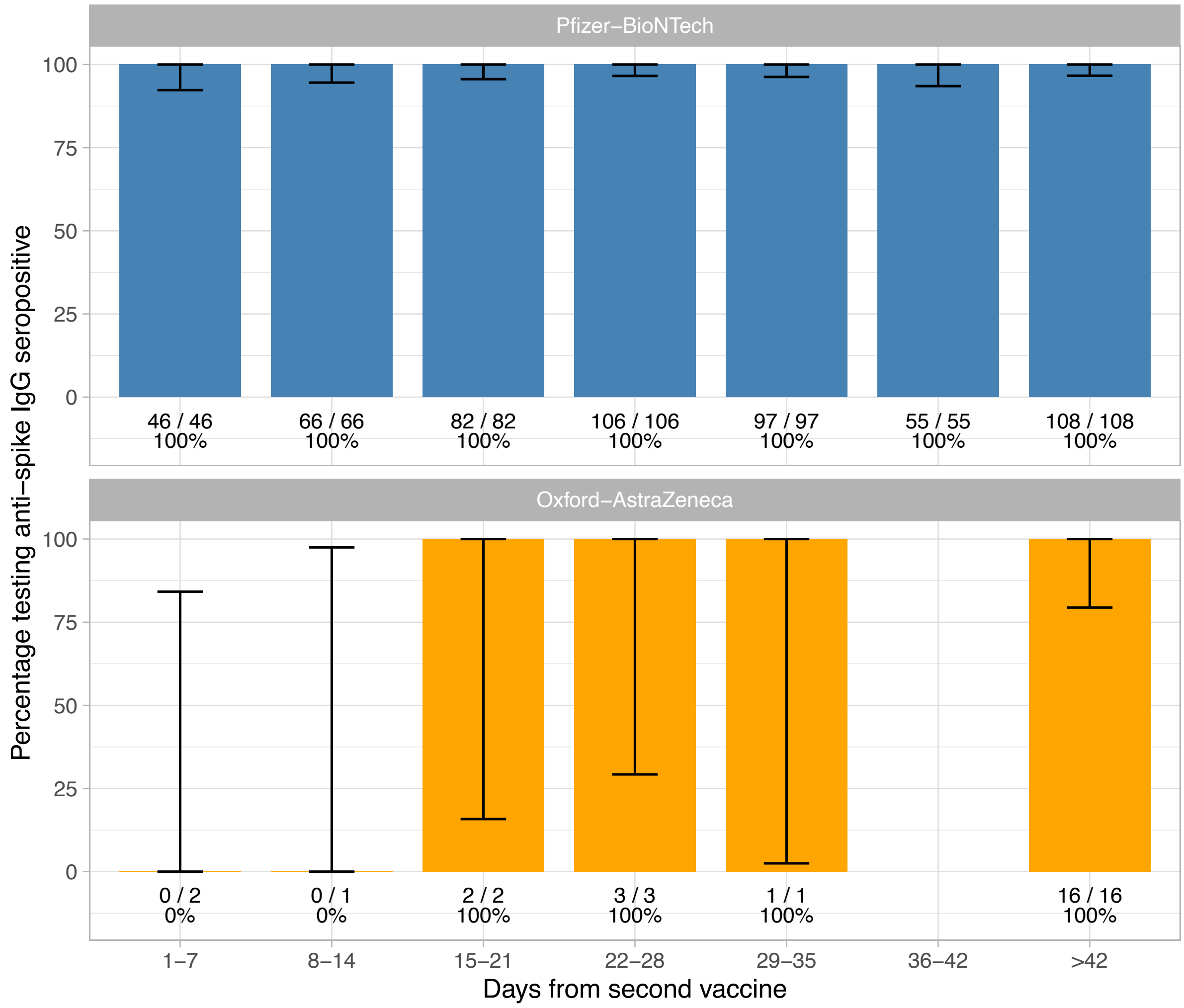

Figure S3. Anti-spike IgG antibody status by vaccine and days since second vaccination.

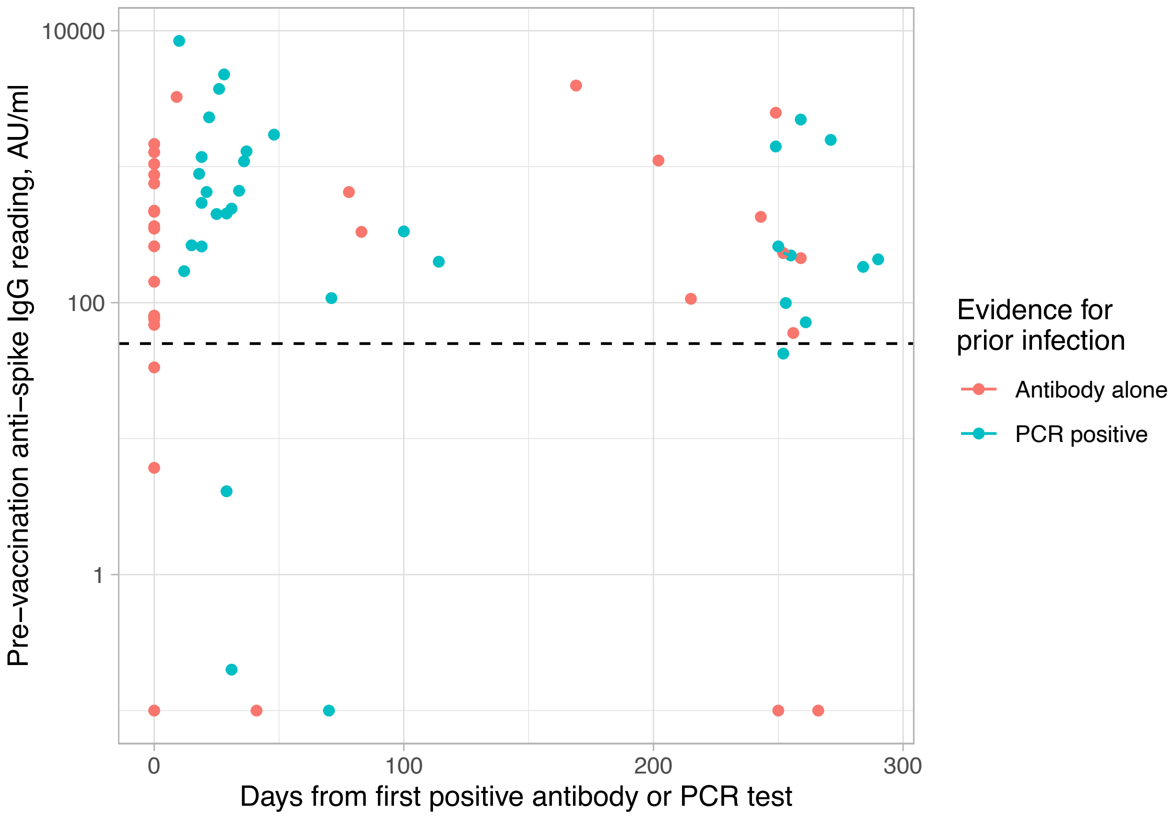

Figure S4. Pre-vaccination Abbott anti-spike IgG antibody levels in 67 previously infected HCWs. Only one pre-vaccination anti-spike result was available per HCW from the Abbott anti-spike assay, as this assay came into use on 11 January 2021. 35 HCWs had a pre-vaccination positive PCR result. For 32 HCWs with only prior positive antibody results, in several cases the antibody result was obtained on another assay such that the point plotted is after time zero on the x-axis. The dashed horizontal line indicates the threshold for a positive antibody result, note the y-axis is shown on a log_{10} scale.

Supplementary tables

Variable	Oxford-AstraZeneca, n = 992[1]	Pfizer-BioNTech, n = 2863[1]	p-value[2]
Gender			0.007
Female	839 (85%)	2,305 (81%)	
Male	153 (15%)	557 (19%)	
Prefer not to say	0 (0%)	1 (<0.1%)	
Age	42 (30, 52)	41 (30, 51)	0.13
Ethnic group			0.002
White	752 (76%)	2,219 (78%)	
Asian	126 (13%)	402 (14%)	
Black	44 (4.4%)	63 (2.2%)	
Other	70 (7.1%)	179 (6.3%)	
Role			<0.001
Admin	125 (13%)	381 (13%)	
Healthcare assistant	137 (14%)	236 (8.2%)	
Junior doctor	21 (2.1%)	122 (4.3%)	
Nurse	291 (29%)	904 (32%)	
Other	333 (34%)	902 (32%)	
Porter/Domestic	23 (2.3%)	40 (1.4%)	
Senior doctor	28 (2.8%)	123 (4.3%)	
Therapist	34 (3.4%)	155 (5.4%)	
[1]n (%); Median (IQR)			
[2]Fisher's exact test; Wilcoxon rank sum test; Pearson's Chi-squared test			

Table S1. Demographics and occupational roles of healthcare workers with antibody measurements available following a first dose of Oxford-AstraZeneca or Pfizer-BioNTech vaccine.

Prior infection status	Vaccine	Occupational role	Age group	Ethnic group	Sex	Highest anti-spike titre before positive PCR, AU/ml	Days from first vaccination to positive PCR
**No prior evidence of infection**	Pfizer-BioNTech	Other	51-60 years	White	Female	644	48
**No prior evidence of infection**	Pfizer-BioNTech	Nurse	≤30 years	White	Male	727	49
**No prior evidence of infection**	Oxford-AstraZeneca	Nurse	≤30 years	White	Female	735	43
**No prior evidence of infection**	Pfizer-BioNTech	Healthcare assistant	41-50 years	Other	Female	946	51
**Anti-spike IgG first detected 8 months prior to vaccination, but anti-nucleocapsid IgG negative; no prior PCR-positive result**	Pfizer-BioNTech	Other	51-60 years	White	Female	1062	37
**No prior evidence of infection**	Pfizer-BioNTech	Junior doctor	31-40 years	Black	Female	1779	46
**No prior evidence of infection**	Pfizer-BioNTech	Nurse	31-40 years	White	Female	2497	46
**No prior evidence of infection**	Pfizer-BioNTech	Porter/Domestic	>60 years	White	Female	8151	70

Table S2. Details of eight healthcare workers with a positive SARS-CoV-2 PCR result following first vaccination. A ninth healthcare worker with a positive PCR result post vaccination is excluded as they also tested PCR-positive prior to vaccination, and the repeat PCR-positive result occurred within 90 days, i.e. was plausibly consistent with residual RNA from the pre-vaccination infection.
